# Relationship of contact angle of spray solution on leaf surfaces with weed control

**DOI:** 10.1038/s41598-021-89382-2

**Published:** 2021-05-10

**Authors:** Renata Thaysa da Silva Santos, Jaqueline Franciosi Della Vechia, Cícero Antonio Mariano dos Santos, Dieimisson Paulo Almeida, Marcelo da Costa Ferreira

**Affiliations:** Department of Plant Production, School of Agricultural and Veterinarian Sciences, São Paulo State University (UNESP), Jaboticabal, 14884-900 Brazil

**Keywords:** Environmental sciences, Invasive species

## Abstract

The weeds are important in agricultural and livestock areas because these plants can cause several damages, especially in the yield. The herbicide pulverization for weed control is the most used, but the efficiency of the control can be dependent the several factors, for example, the correct chose the herbicide and the mixture or not with adjuvant. This study aimed to evaluate the contact angle of herbicide solution droplets associated with adjuvant when deposited on the leaf surface of different weed species and their relationship with chemical control. For the contact angle experiment, the design was completely randomized, with four repetitions, while for the control experiment, a randomized block design was used, both experiments were arranged in a factorial (4 × 2 + 1) design. Factor A corresponded to four spray solutions containing the herbicide no addition of adjuvants and herbicide associated with adjuvants (vegetable oil, mineral oil, and lecithin), factor B to two herbicide dosages, and additional treatment corresponded to water. The contact angle was determined in six weed species: *Crotalaria incana*, *Lantana camara*, *Ipomoea grandifolia*, *Asclepias curassavica*, *Sida obtusifolia,* and *Ricinus communis*, on the adaxial and abaxial surface of each species, and an artificial surface. For the weed control experiment was used two weed species: *C. incana* and *L. camara*. The multivariate analysis allowed the understanding of the behavior of the contact angle of the different groups on the natural and artificial surfaces, due to the formation of factors. For all plants, except for the abaxial surface of *I. grandifolia* and the adaxial surface of *A. curassavica,* the association of herbicide and adjuvants reduced contact angle on the surfaces. The chemical control resulted in an indirect relation with contact angle, where smaller contact angles of the herbicide solution resulted in a higher percentage of plant intoxication. Therefore, for this situation, it is recommended to use the herbicide aminopyralid + fluroxypir associated with lecithin.

## Introduction

Weeds are considered one of the main degradation factors of pastures in the world due to competition with forage plants for light, space, and nutrients^1^. Moreover, some of them are toxic to ruminants, several plants have substances that cause damage to the brain and/or liver failure, causing the death of the animals. Due to this, many farmers have problems in pastures because of the presence of weed, in addition to decreasing forage productivity, it also can cause a decrease in the herd due to the death of the animals^2,3^. Several species of weeds can occur in pastures, mainly species from the families Asclepiadaceae, Caesalpinoideae, Chenopodiaceae, Euphorbiaceae, Leguminoseae, and Verbenaceae^4–8^.

The main method used to control these weeds is chemical control^9^. Although it is an effective, fast, and low-cost method, chemical control can cause damage to forage plants. Therefore, is important to use selective herbicides to reduce this problem. A selective herbicide used on pasture is the aminopyralid + fluroxypyr commercial formulation, which belongs to the group auxin mimic, that cause epinasty in leaves and stem, consequently the death of the plant^10^. The use of this herbicide in the field is often associated with adjuvants to increase the efficacy of the herbicide. Adjuvants may alter the physicochemical properties of the spray solution, such as viscosity, contact angle, hydrogen potential (pH), and electrical conductivity^11–13^. The reduction of the contact angle that a drop forms on a surface may improve the penetration and absorption of the products by plants^14^.

The contact angle is used to characterize the droplets deposited on the solid surface, considering the interaction factors of the liquid spreading on the surface and the area covered by spraying. A surface is considered hydrophilic when the contact angle is less than 90°, otherwise, it is considered hydrophobic. Some surfaces can show superhydrophobicity when the contact angle of a drop on the surface is above 160°^15^. Therefore, the weed leaf surface will determine the contact angle of the herbicide solution drop on the surface. Menendez et al.^16^ found that in *Avena fatua* L. the contact angle of a solution of glyphosate mixed with adjuvants was greater than in plants of *Lolium rigidum* Gaud. and *Alopecurus myosuroides* Huds. Another factor that will determine the effectiveness of mixtures of herbicides with adjuvants is the type of adjuvant used since the physical and chemical characteristics of these products can interact in different ways with the leaf surface^17^. Thus, for herbicides that depend on the quality of plant coverage to perform their effectiveness, the low product retention provided by hydrophobic surfaces can result in failures in weed control.

The spraying process is extremely complex because it includes droplet formation, retention of droplets sprayed on the plant surface, and spreading on the surface, as well as permeation of the active ingredient through the cuticle of the plant until it effectively reaches the target^18^. Because of that, this study aimed to evaluate the contact angle of droplets of herbicide associated with adjuvants deposited on the leaf surface of different pasture weed species and their relationship with the weed control.

## Results

The results were divided into two factors, both were an independent process that was occurring on the surfaces, together, the two factors explained 60.2% of the total variation of the original data. Factor 1, which consists of the contact angle and the weed control, represent 60.1% of the data, and Factor 2, which consists of the contact angle of the abaxial surface of *I. grandifolia* and the adaxial surface of *A. curassavica*, represent 0.9% of the total variation (Table 1).

**Table 1:** Result of the analysis of factors containing the first two factors (processes) with their respective factorial loads that represent the correlation coefficients between the foliar surfaces and control and each Factor.

Variable	Factor 1	Factor 2
	60.10%*	0.9%*
Artificial surface	**0.98**	0.02
*L. camara* Adaxial	**0.75**	0.24
*L. camara* Abaxial	**0.68**	−0.04
*C. incana* Adaxial	**0.95**	0.00
*C. incana* Abaxial	**0.96**	−0.02
*I. grandifolia* Adaxial	**0.81**	0.39
*I. grandifolia* Abaxial	0.45	**0.54**
*S. obtusifolia* Adaxial	**0.88**	0.18
*S. obtusifolia* Abaxial	**0.94**	0.18
*R. communis* Adaxial	**0.52**	0.26
*R. communis* Abaxial	**0.85**	0.18
*A. curassavica* Adaxial	−0.12	**0.53**
*A. curassavica* Abaxial	**0.73**	0.02
Control *C. incana* 5DAP	−**0.50**	0.40
Control *C. incana* 11DAP	−**0.77**	0.49
Control *C. incana* 16DAP	−**0.92**	0.25
Control *L. camara* 5DAP	−**0.51**	0.58
Control *L. camara* 11DAP	−**0.84**	0.19
Control *L. camara* 16DAP	−**0.95**	0.02
**Interpretation**	Surface and control	*Ipomoea grandifolia* (abaxial) and *Asclepias curassavica* (adaxial)

*Value relative to the percentage of variation of the original set of data retained by the respective factors. Load values in bold (> 0.50 in absolute value) were considered in the interpretation of the factor. Artificial surface = Parafilm; DAP = Days after pulverization.

The process contained in the contact angle and weed control is the most important for this study since it is derived from the higher eigenvalue and has a higher percentage of explanation (60.1%), and the variables that contribute the most are represented by artificial surface (0.98), lantana adaxial and abaxial face (0.75 and 0.68, respectively), shakeshake adaxial and abaxial face (0.95 and 0.96, respectively), morning glory adaxial face (0.81), sicklepod adaxial and abaxial face (0.88 and 0.94), castor bean adaxial and abaxial face (0.52 and 0.85), bloodflower milkweed abaxial face (0.73), control in shakeshake 5, 11 and 16 DAA (−0.50, −0.77 and −0.92, respectively), control in lantana 5, 11 and 16 DAA (−0.51, −0.84 and −0.95, respectively). Furthermore, according to the signs of the factorial loads, the contact angle and weed control factor are directly and strongly correlated with the surfaces, because the contact angle showed the same positive sign for both surfaces, as well as to the control shakeshake and lantana showed the same negative sign.

**Figure 1:**
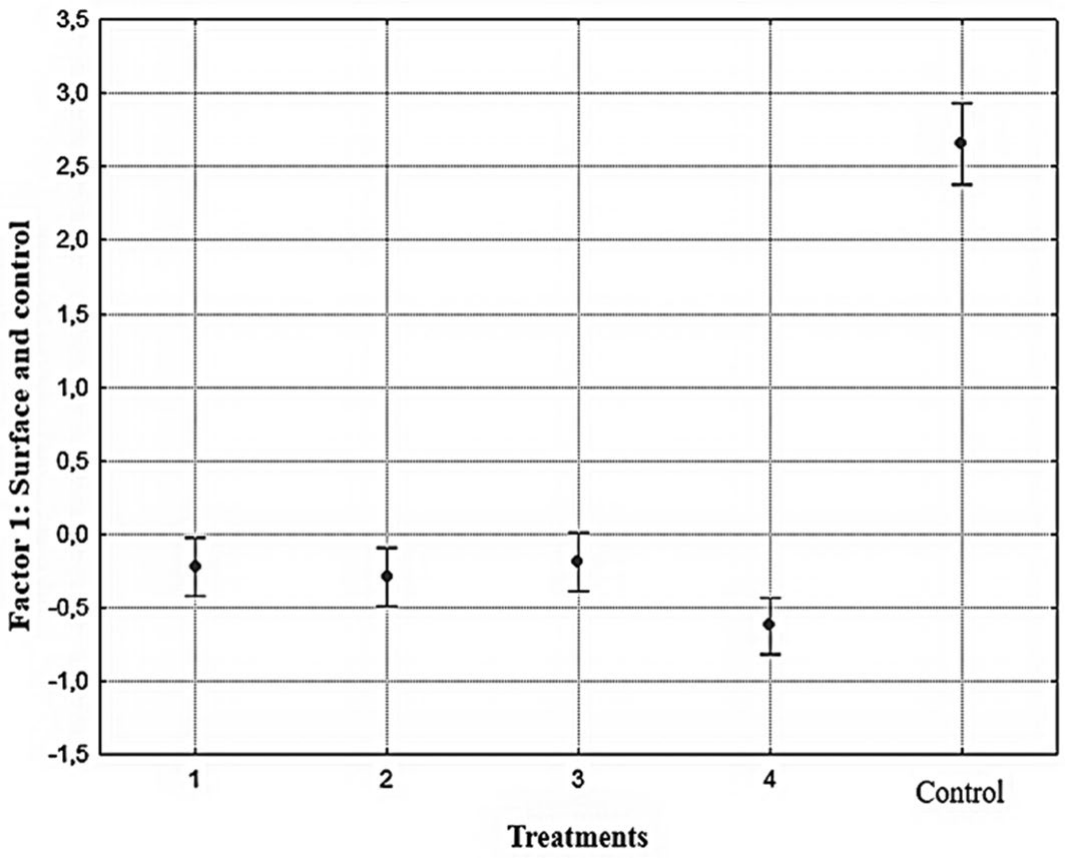
Graphical representation with the scores of Factor 1 (surface and control) as a function of the evaluated samples. Vertical bars represent confidence intervals of 0.95 (F = 109.58; p = 0.0001). Spray solution: 1-No adjuvant; 2-herbicide associated with vegetable oil; 3- herbicide associated with mineral oil; 4-herbicide associated with lecithin, and Control.

**Figure 2:**
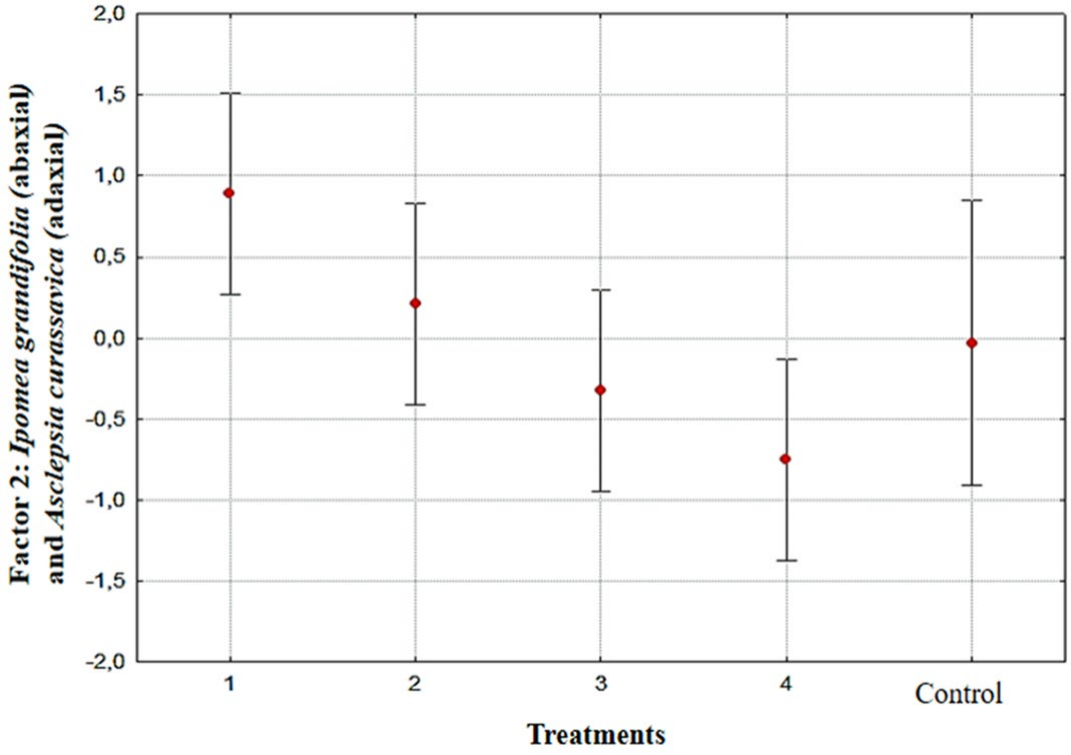
Graphical representation with Factor 2 (*Ipomoea grandifolia* (abaxial) and *Asclepias curassavica* (adaxial) scores) as a function of the evaluated samples. Vertical bars represent confidence intervals of 0.95 (F = 4.036, p = 0.009). Spray solution: 1-No adjuvant; 2-herbicide associated with vegetable oil; 3-herbicide associated with mineral oil; 4-herbicide associated with lecithin, and Control.

Considering that the factors are orthogonal (uncorrelated), the processes retained in the contact angle, weed control (Factor 1), and the factor morning glory abaxial surface, bloodflower milkweed adaxial surface (Factor 2) are considered independent. Thus, the analysis was performed with the scores of the contact angle and control factor (Fig. 1) and the morning glory abaxial surface factor, and bloodflower milk weed adaxial surface (Fig. 2). A significant difference (F = 109.58; p = 0.0001) was found between the treatments when the surfaces and weed control were evaluated (Factor 1). There were differences between the spray solutions and water treatment.

For factor 2 there was also a difference (F = 4.036; p = 0.009) when the plants were evaluated together. The spray solution did not differ from the control. When the spray solutions were compared, there was a difference only between the herbicide spray solution with no lecithin and the herbicide spray solution with lecithin (Fig. 2).

Correlating with the results of the univariate analysis, it is possible to observe that the separation of factors is related to the result obtained from the contact angle of the bloodweed milkweed and morningglory surfaces, because, for bloodflower milkweed, the treatments and control were not significant (p > 0.05), with no significance for the dosage and adjuvant interaction (p > 0.05). The two experiments to control the weeds were complementary, did not differ from each other (p > 0.05).

**Figure 3:**
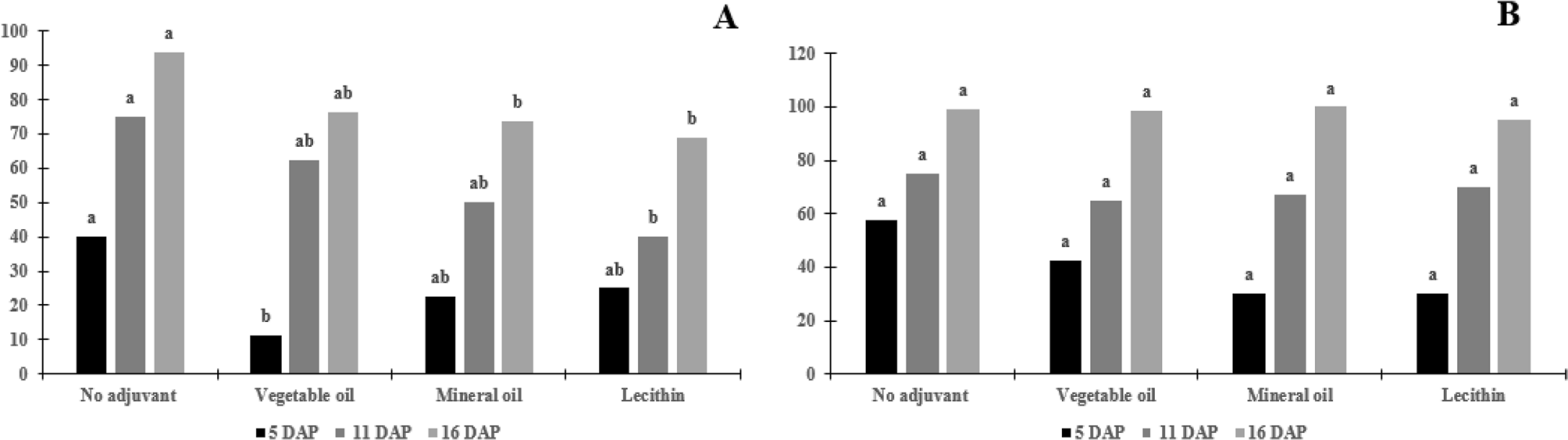
Percentage of control of *Crotalaria incana* L. plants after herbicide solution spraying. (**A**) Aminopyralid + fluroxypyr at 155.3 L of active ingredient ha^−1^. (**B**) Aminopyralid + fluroxypyr at 360.6 L of active ingredient ha^−1^. Equal letters within the evaluation days, the adjuvants do not differ from each other by the Tukey test (p > 0.05).

**Figure 4:**
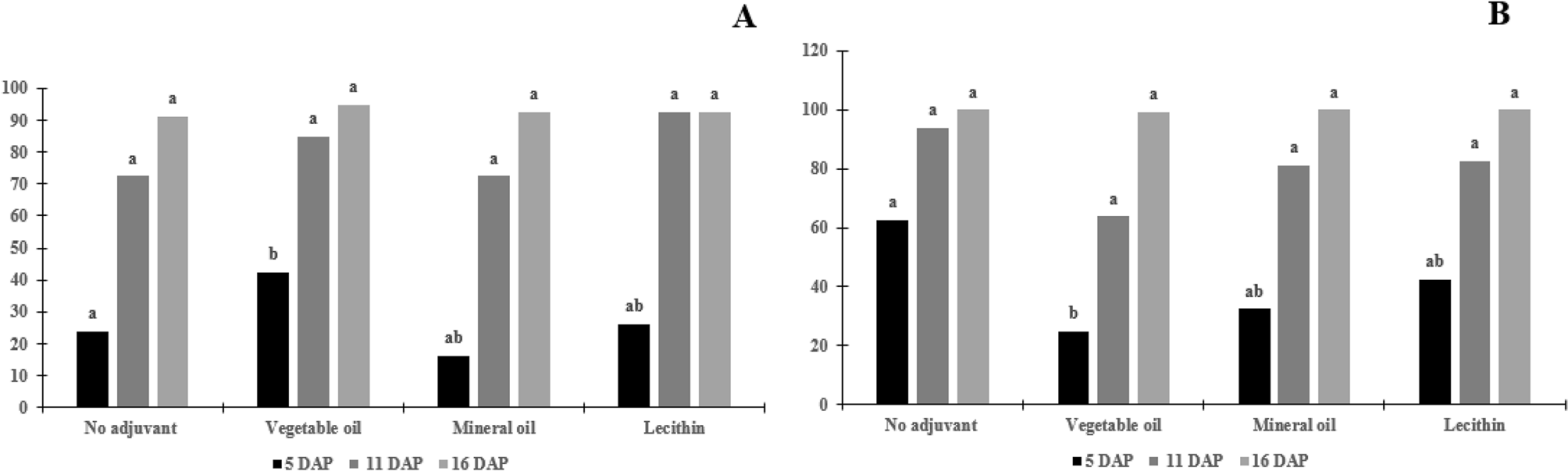
Percentage of control of *Lantana camara* L. plants after herbicide solution spraying. (**A**) Aminopyralid + fluroxypyr at 155.3 L of active ingredient ha^−1^. (**B**) Aminopyralid + fluroxypyr at 360.6 L of active ingredient ha^−1^. Equal letters within the evaluation days, the adjuvants do not differ from each other by the Tukey test (p > 0.05).

For lantana on the adaxial surface, the control treatment differed from the treatments (p = 0.001), however, the spray solution, doses, and interaction were not significant (p > 0.05, 0.0844, and 0.0616, respectively) (Table 2). For the abaxial surface, the spray solutions and the interaction of the factors were not significant (p = 0.0535 and 0.1353, respectively). However, the herbicide doses were statistically different (p > 0.0001) (Figs. 3, 4).

**Table 2:** Average and standard deviation of the contact angle (°) after drop deposition in surfaces of the leaves of the weeds.

Variable	Dose	No adjuvant	Vegetable oil	Mineral oil	Lecithin	Control
Artificial surface	155.3 L ha^−1^	70.55 a	72.38 a	70.40 a	66.33 b	109.91*
	310.6 L ha^−1^	65.91 a	64.60 a	64.48 a	62.11 b	109.91*
*L. camara* Adaxial	155.3 L ha^−1^	59.94 a	51.35 a	51.57 a	42.35 a	74.65*
	310.6 L ha^−1^	50.54 a	47.22 a	58.30 a	32.51 a	74.65*
*L. camara* Abaxial	155.3 L ha^−1^	70.02 a	68.97 a	75.45 a	72.03 a	87.82*
	310.6 L ha^−1^	60.14 a	54.43 a	49.51 a	60.89 a	87.83*
*C. incana* Adaxial	155.3 L ha^−1^	82.06 ab	80.95 b	86.28 a	84.33 ab	146.62 *
	310.6 L ha^−1^	78.72 a	71.53 b	78.22 a	76.26 ab	146.22*
*C. incana* Abaxial	155.3 L ha^−1^	76.72 b	80.52 ab	87.86 a	74.89 b	148.2*
	310.6 L ha^−1^	75.13 ab	75.42 ab	79.88 a	74.41 b	148.32*
*I. grandifolia* Adaxial	155.3 L ha^−1^	65.00 a	62.36 ab	52.10 b	43.35 c	80.95*
	310.6 L ha^−1^	58.76 a	50.95 b	50.34 b	41.42 c	80.95*
*I. grandifolia* Abaxial	155.3 L ha^−1^	54.18 ab	61.09 a	38.81 b	36.88 b	67.33*
	310.6 L ha^−1^	54.49 ab	40.58 b	63.85 a	46.43 ab	67.33*
*S. obtusifolia* Adaxial	155.3 L ha^−1^	102.38 a	93.48 b	90.59 b	74.30 c	132.88*
	310.6 L ha^−1^	71.74 b	85.09 a	74.72 b	58.37 c	132.88*
*S. obtusifolia* Abaxial	155.3 L ha^−1^	107.38 a	88.20 b	84.44 b	82.09 b	177.79*
	310.6 L ha^−1^	94.58 a	90.72 ab	81.19 b	66.35 c	177.79*
*R. communis* Adaxial	155.3 L ha^−1^	54.69 a	47.59 a	43.76 ab	33.15 b	64.42*
	310.6 L ha^−1^	40.62 b	41.55 b	53.12 ab	57.21 a	64.42*
*R. communis* Abaxial	155.3 L ha^−1^	63.60 a	61.78 a	57.73 a	43.01 b	81.5*
	310.6 L ha^−1^	49.26 a	36.92 b	52.47 a	46.58 a	81.5*
*A. curassavica* Adaxial	155.3 L ha^−1^	48.81 a	46.20 a	42.11 a	40.51 a	43.93*
	310.6 L ha^−1^	40.83 a	52.92 a	37.95 a	50.49 a	43.94*
*A. curassavica* Abaxial	155.3 L ha^−1^	57.47 a	48.21 a	52.08 a	49.54 a	68.77*
	310.6 L ha^−1^	51.71 a	56.34 a	53.80 a	48.07 a	68.77*

Equal letters on the line do not differ from each other by the Tukey’ test (p > 0.05). *The control was different from the treatments by the Tukey’ test (p > 0.05).

**Figure 5:**
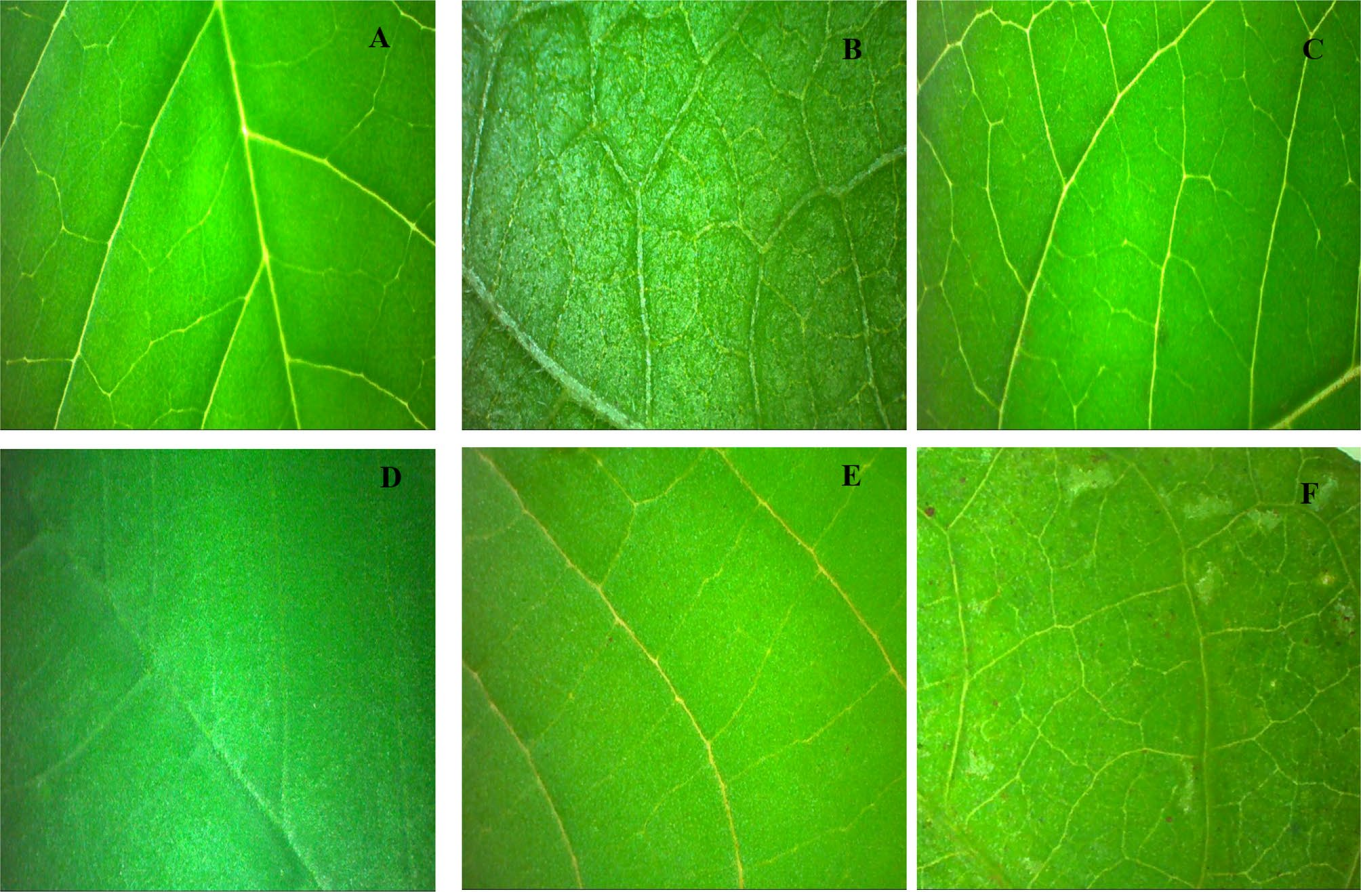
Surface adaxial in plants of the *Crotalaria incana* L. (**A**), *Lantana camara* L. (**B**), *Asclepias curassavica* L. (**C**), *Senna obtusifolia* (L.) H.S.Irwin & Barneby (**D**), *Ricinus communis* L. (**E**) and *Ipomoea grandifolia* (Dammer) O'Donell (**F**) taken on the Stereoscopic Microscope with × 1.0.

**Figure 6:**
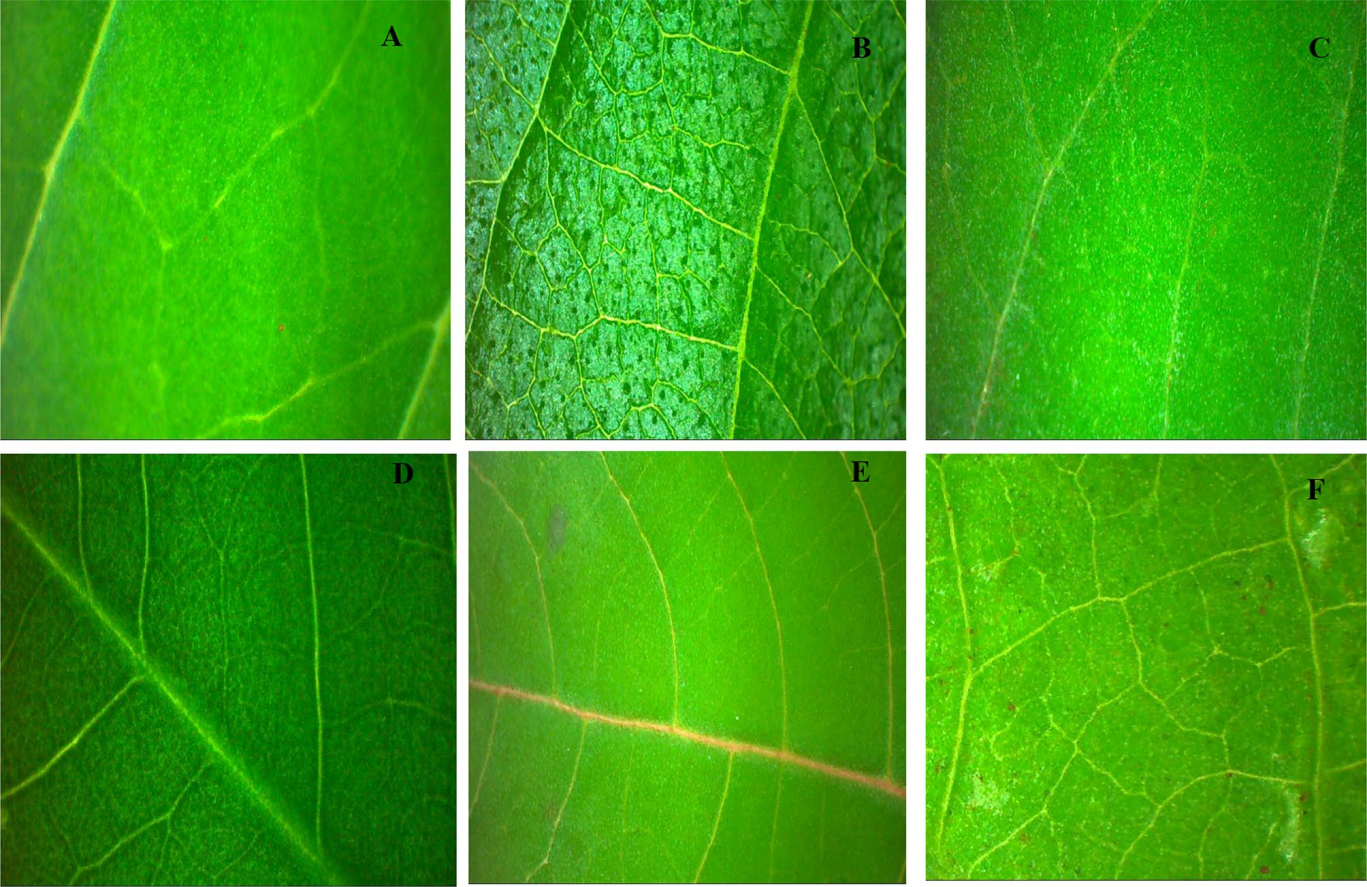
Surface abaxial in plants of the *Crotalaria incana* L. (**A**), *Lantana camara* L. (**B**), *Asclepias curassavica* L. (**C**), *Senna obtusifolia* (L.) H.S.Irwin & Barneby (**D**), *Ricinus communis* L. (**E**) and *Ipomoea grandifolia* (Dammer) O'Donell (**F**) taken on the Stereoscopic Microscope with × 1.0.

The contact angle of the shakeshake adaxial surface did not differ for spray solutions (p = 0.666), dose and control factors versus the treatments were significant (p > 0.0001 and 0.0001), but for interaction, there was no significance (p = 0.5327) (Table 2). The abaxial surface, the spray solution, doses, and the control treatment versus the treatments were significant (p = 0.0056, 0.0428, and 0.0001), but the interaction was not significant (0.4453) (Table 2). For sicklepods adaxial and abaxial surfaces, doses, spray solution, control versus treatments and interaction were significant (p > 0.0001). The surfaces of the species shakeshake and sicklepod showed higher values to the control, the abaxial surface sicklepod presented the contact angle value of approximately 177° (Table 2; Figs. 5 and 6), for shakeshake the addition of vegetable oil decreased the contact angle on the adaxial surface at any dose, and for sicklepod the addition of lecithin resulted in the lowest contact angle (Table 2).

For the castor bean, on the adaxial surface, the spray solution and doses were not significant (p = 0.06126 and 0.1761, respectively), the control treatment and the interaction were significant (p > 0.0001), but for the abaxial surface the spray solution, dose, interaction, and control treatment were significant (p > 0.0001). The morningglory on the adaxial surface showed a significant difference between the spray solution, doses, control treatment (p = 0.0001, 0.0072, and 0.0001) but it was not significant for the interaction (p = 0.2283), on the abaxial surface, the spray solution and the doses were not significant (p = 0.0755 and 0.3025), but the interaction and the control treatment were significant (p = 0.0007 and 0.0018). For morningglory the addition of lecithin resulted in the lowest contact angle on the adaxial surface, for the abaxial addition of mineral oil and lecithin presented the lowest values, for the castor beans the lecithin resulted in the lowest contact angle on the adaxial surface for the dose of 155.3 L ha^−1^, however, at a dose of 310.6 L ha^−1^, the spray solution without adjuvant or mineral or vegetable oil showed lower values of contact angle.

For the milkweed bloodflower, on the adaxial surface, the sprays solution, doses, interaction, and control treatment were not significant (p = 0.1320, 0.6804, 0.0848, and 0.800, respectively), the abaxial surface, the sprays solution, doses, and interaction were not significant (p = 0.2016, 0.7371 and 0.8916, respectively), so the contact angle values were statistically similar to each other. However, its species presented a lower average value of the contact angle (43.93°) compared to other plants (Fig. 5 and 6).

The standard surface (Parafilm) sprays solutions, control treatment and doses were significant (p > 0.0001) but there was no significance for interaction (p = 0.2077), thus, regardless of the dose, the addition of lecithin resulted in the lowest contact angle of the drops.

In the weed control experiment, shakeshake and lantana plants showed rapid damage caused by the herbicide aminopyralid + fluroxypyr (Fig. 3 and 4). For shakeshake, at 5 DAP the spray solution and dose were not significant (p > 0.05), the control treatment versus the treatments and the interaction was significant (p = 0.0019 and 0.0149, respectively), the interaction was significant only in mineral oil in that the level of control was lower at the dose of 155.3 L ha^−1^. At 11 DAP the spray solution was not significant (p > 0.05), but the doses, the interaction, and the control treatment versus the treatments were significant (p = 0.0009, 0.0413, and 0.0001, respectively), the interaction was significant because there were differences in lecithin at the control level, in which the dose of 155.3 L ha^−1^ resulted in the lowest level of control. In the last evaluation at 16 DAP, the spray solution was not significant (p > 0.05), dose, interaction, and control versus treatments were significant (p = 0.0001, 0.0353 and 0.001), the interaction was significant for the spray solutions that had the addition of an adjuvant, in which the highest level of control was observed at the dose of 310.6 L ha^−1^ (Fig. 3 and 4).

The level of lantana control at 5 DAP showed significance for spray solution (p = 0.0257) and control versus treatments (0.007), the dose and interaction were not significant (p > 0.05), at 11DAP the spray solution, dose, and interaction were not significant (p = 0.1377, 0.0706 and 0.6540, respectively) there was only significance for control versus treatments (p = 0.0001). At 16 DAP the spray solution, dose, and interaction were not significant (p = 0.4703, 0.1734, and 0.4210, respectively), there was significance for the control versus treatments (p = 0.0004) (Fig. 3 and 4).

In general, at 5 DAP, both species showed symptoms of twisting of the leaves next of the apical region was observed differences between treatments with no adjuvant and the doses used. The dose of 310.6 L ha^−1^ showed a better percentage of control in both species, and independent of the treatments used (Fig. 3 and 4).

At 16 DAP, lantana plants showed 100% control independent of the dose used, but the shakeshake plants had control of close to 80% independent of the dose (Fig. 4). Shakeshake plants have a lower percentage of control due to the surface being more hydrophobic to the surface of the lantana, hydrophobicity can cause the drops to ricochet, with no drops being deposited, therefore, the product does not absorb (Fig. 3).

The addition of adjuvants to the spray solution did not result in differences between the treatment without addition, despite the decrease in the contact angle with the addition of the herbicide. In the shakeshake, a higher percentage of control was observed in the treatment without the addition of an adjuvant, in the dose of 155.3 L ha^−1^, in the dose of 310.6 L ha^−1^ with the addition of lecithin resulted in a higher percentage of control (Fig. 3). For lantana, the addition or not of the adjuvant did not result in different control percentage values, independent of the dose, that is, only the herbicide was necessary to obtain 100% control of the plants, this is due to the lower angle value of contact compared to shakeshake (Fig. 4).

## Discussion

The surfaces of the plants showed similarities in the dynamics of spreading the droplets. The contact angle of the water was different from the treatments, showing a greater contact angle, except for the bloodweed surface, in which the values of water and spray solutions did not result in statistical differences. Singh, Orsenico, and Shah^19^ studying several adjuvants observed that the addition of adjuvant in the distilled water reduced the contact angle and the surface tension of the sprayed drops, corroborating what was found in this study.

In general, the surfaces presented in this study have an affinity with the spray solution. This affinity may be due to the wax composition of the leaves, for example, the leaves of the castor bean species have oil droplets incorporated into a mass of phenolic compounds, inside the vacuole of the epidermal cell^16^, which can contribute to the spreading of the drops in these surfaces. Other characteristics can also influence the spread of the drops, for example, the number of stomata. The high value of the contact angle found for the shakeshake may be since this plant has many stomata on the adaxial side^20,21^. Sicklepod presents a star-shaped epicuticular wax, with trichomes on the adaxial face and irregularly dispersed on the abaxial face^22^. Lantana also has stomata on the adaxial and abaxial surfaces^23^, but this characteristic did not interfere with spreading, that is, the spray solution showed more affinity with the lantana surface compared to the shakeshake, this indicates that although the stomata influenced the spread of the drop on the surface, another factor was responsible for the high value of contact angle found in this study. Therefore, the presence of stomata did not influence the differences in the contact angle of the drops on the surfaces, but it must be related to other factors, such as the composition of the epicuticular wax.

According to the classification of Tang^15^, shakeshake and sicklepod can be characterized as superhydrophobic surfaces (Table 2). Therefore, there is a need to add adjuvants to the spray solution, since, when applied in pasture areas with a high flow of this species, the non-addition of the adjuvant can compromise the efficiency of chemical control. However, it is also necessary to observe the type of herbicide formulation to assess the need for the addition and the type of adjuvant to be used, in this study a herbicide with a water-in-oil (WO) emulsion formulation was used, which can clarify the results the contact angle found for some surfaces, as an example, for the castor adaxial surface (Table 2). In addition, in commercial formulations, information on the composition of the aggregates is not available, that is, it is not known about the quantity and type of emulsifiers that exist in a commercial formulation, in general, commercial formulations are developed to facilitate adherence drop on the leaf surfaces of plants, therefore, in some situations, it is not necessary to add adjuvants.

The lowest values of contact angle found on castor bean, morningglory, and bloodweed surfaces should be due to the composition of the surfaces, resulting in differences in the spreading dynamics. The bloodweed contains fixed oils, flavonoids, phenols, quinine, tannins, ascorbic acid, terpenoid, sugars, xanthoprotein, saponin, and steroids^24^, leaf waxes are dominated by long-chain aldehydes, trichomes, and wax crystals, with lower stomatal density^25^. The morningglory surface is composed of primary and secondary alcohols and esters, hydrophilic compounds, which allows greater surface affinity with the droplets^26,27^, however, plants of the genus ipomea have stomata and trichomes in both surfaces (adaxial and abaxial)^27^. Drops on hydrophilic surfaces, such as plants of the Ipomoea genus, have a greater coverage area and shorter evaporation time when compared to hydrophobic surfaces, in this study represented by the hydrophobic surfaces of the shakeshake^28^. Thus, the bloodweed's adaxial surface was classified as hydrophilic^15^, and therefore no differences were observed between adjuvants and doses. Therefore, in pasture areas where bloodweed infestation predominates, the use of adjuvants is not necessary.

In this study, an indirect relationship was observed between the surface of plants and chemical control. Thus, when the drop contact angle is smaller, the intoxication effect of the plants is greater. The herbicide used aminopyralid + fluroxypir belongs to the auxin-mimicking mechanism of action, herbicides of this mechanism are selective, not affect forage plants (Poaceae family)^10^. At 5 DAP the plants showed symptoms of poisoning by the herbicide, regardless of the dose used. In studies carried out in the chemical control of *Vismia guianensis*, the symptoms of yellowing of the leaves were observed, with a reduction in the chlorophyll content 15 days after spraying^29^, different from what was observed in this study. Although this herbicide can cause the death of plants within five weeks after spraying, in this study we observed the critical symptom in the first five days. The speed for the onset of maximum intoxication may be due to the addition of adjuvants that allowed a better dispersion of the droplet and consequently better penetration of the product. Because, when there is no presence of surfactants, the droplets remain spherical, as with water, resulting in a spreading peak of 0.1%, while, for pesticide drops, it can reach a peak of 1%^30^.

The speed at which symptoms appear can also be related to the herbicide doses used, in isolated situations the dose of 310.6 L of active ingredient ha^−1^ resulted in a higher percentage of control, but at the end of the experiment, there was no difference between doses. Differences between the adjuvants were observed, mainly in solutions sprayed on shakeshake and lantana plants, at 5DAP the vegetable oil showed a lower percentage of control, not being observed for the other days of evaluation. The addition of mineral or vegetable oils to the spray solution is recommended for the control of Shakeshake^20^. Although the literature suggests the addition of this type of adjuvant, in this research, it was found that choosing to add lecithin to the spray solution or not to add adjuvant resulted in a higher rate of symptom when compared to oil-based adjuvants.

The adjuvants based on vegetable and mineral oil present different types of extraction, in this process, it can interfere in the affinity with the leaf surface, the vegetable oil to be originated from the plants, affinity with the vegetable surface is expected. Mineral oils come from a fraction of the distillation of petroleum, while the vegetable comes from extraction using solvents, are hydrocarbons with 16 or 18 carbons, but before they are purified to remove resins and mucilage, and the surface tension and angle properties of contact are related to the quality and quantity of emulsifiers added to the formulation^31^. The adjuvant Lecithin is a mixture of phosphatidylcholine and propionic acid, commonly used to decrease drift, due to the presence of propionic acid also causes a reduction in the pH of the spray solution. In a study carried out with the herbicide 2,4-D, it was found that the addition of this adjuvant, regardless of the tip model used, reduced the herbicide drift by 4.9%^32^.

However, the relationship between the contact angle and control is inversely proportional, that is, when the contact angle decreases, the percentage of control of the species increases. In an infested pasture area, there is a huge diversity of species that in many situations makes it difficult to choose the herbicide and adjuvant. Therefore, one of the ways to achieve effective weed management in pastures is to monitor the existing flora, as well as the frequency with which weeds occur. In addition, the choice of adjuvants must be related to the type of herbicide formulation and the most frequent plants in the area. It is important to note that other factors may interfere with the contact angle of the droplets formed on a surface, such as natural hygroscopic composition, leaf surface moisture, stomatal activity^30^. Knowledge about the leaf morphology of these plants will also assist in the effective management of weeds, including the correct use of spraying techniques. In addition, it is important to demonstrate that the results obtained in this study are directed only to the spraying of hormonal herbicides, therefore studies are needed that observe the behavior of other herbicide molecules with other mechanisms of action, that is, other tank mixtures.

## Conclusions

The reduction of the contact angle of drops of aminopyralid + fluroxypyr solution on leaf surfaces depends on the use of an adjuvant. The value of the contact angle has an indirect relationship with the chemical control of the weeds. When the contact angle is smaller, the percentage of intoxication is higher. The surfaces of the plant were classified as hydrophobic. Due to the great diversity of weed flora in pasture areas, it is recommended to add lecithin in the solutions of aminopyralid + fluroxypyr.

## Methods

Two experiments were carried out, one to verify the contact angle of herbicide and adjuvant mixtures on plant and artificial surfaces, and the other to evaluate weed control. The contact angle experiment was carried out in a completely randomized design, and the weed control experiment, in a randomized block design. Both used a 4 × 2 + 1 factorial scheme, with four replicates. Factor A corresponded to four spray solutions composed of herbicide no addition of adjuvants and herbicide associated with adjuvants (vegetable oil, mineral oil, and lecithin based), factor B corresponded to two herbicide dosage (155.3 L ha^−1^ and 310.6 L ha^−1^ a.i.) and additional treatment was spraying only water. The herbicide used was Aminopyralid + fluroxypyr (commercial name Dominum, company Corteva), and three adjuvants: (1) Fatty acid esters with glycerol (commercial name Veget’oil, company Oxiquímica Agrociência), (2) Aliphatic hydrocarbons (commercial name Argenfrut, company Agrovant Comércio de Produtos Agrícolas) and (3) Mixture of phosphatidylcholine and propionic acid (commercial name LI-700, company DE SANGOSSE Agroquímica), all adjuvants were added to the spray solution in the ratio of 0.3% v v^−1^. The dosages used were based on the recommendation of the instruction insert of the commercial, to evaluate the minimum and maximum recommended dosages.

## Contact angle experiment

The contact angle was determined using the Contact Angle System OCA 15-Plus (Dataphysics) tensiometer fitted with a high-resolution, time-resolution digital camera and SCA20 software (https://www.dataphysics-instruments.com/products/oca/software/) for automation and processing of images. To determine the variables, the droplets were formed in a 500 μL Hamilton precision syringe, and the droplet release rate was set at 3 μl s^−1^ for all treatments. Evaluations were made for six species: shakeshake (*Crotalaria incana* L.), lantana (*Lantana camara* L.), morning glory (*Ipomoea grandifolia* (Dammer) O'Donell), bloodflower milk weed (*Asclepias curassavica* L.), sicklepod (*Senna obtusifolia* (L.) H.S.Irwin & Barneby), and castor bean (*Ricinus communis* L.), on the adaxial and abaxial surface of each species, and all the methods followed relevant guidelines and regulations, for involving the collection of plant material in the study protocols. Besides, evaluations were made on an artificial surface (a water-resistant, paper-thin plastic paraffin film marketed as Parafilm M).

The weeds were cultivated in a greenhouse; the pots were filled with substrate, composed of sand, bovine manure, and soil, at a 3:1:3 ratio. The collected leaves were sectioned into longitudinal rectangles of approximately 5 cm × 1 cm. These sections were arranged horizontally on their stretchers to reduce undulations that could compromise the leaf structure and the capture of images for contact angle readings. The images were evaluated every second for 60 s after the deposition of each droplet on the leaf surface. For data analysis, images produced at five seconds were selected. The temperature and relative humidity average during the readings were 74.88°F and 60%, respectively.

## Weed control experiment

In the weed control experiment, the herbicide solution with or without adjuvant was sprayed at the post-emergence of two species were selected that represent hydrophobic and hydrophilic surfaces, most frequently in pasture areas and toxic to animals: shakeshake and lantana at 65 days after sowing and planting the cuttings, respectively. The spraying was performed with a CO_2_ pressurized research sprayer mounted on a quadricycle, fitted with a bar at a height of 60 cm from the plants, with support for three spray tips spaced at 0.5 m each other, operating at a constant pressure of 1.5 bar, and speed of 3.58 mm per hour. The spray tip used was TTI 11,003, with a tip flow of 0.72 L min^−1^, classified as an ultra-thick droplet. The application volume was 150 L ha^−1^. The atmospheric conditions were measured at the time of application using a digital thermohygrometer, for each treatment applied. Application conditions were average temperature = 77.46 °F, humidity = 85.12%, wind speed = 0.018 mi per hour. For validation of the results, the experiment was repeated, thus, the first repetition happened in April and the second repetition in November. The conditions were used: the average temperature at application = 90.0°F, humidity = 65.12%, wind speed = 0.018 mi per hour.

The pots were kept in an area adjacent to the greenhouse of the Department of Plant Protection and irrigated daily. Plant intoxication assessments were carried out using a scale; scores were assigned for a percentage of injury caused by the herbicide in the plant area, where 0% represents no injuries and 100% represents plant death^33^. Evaluations occurred at 5, 11, and 16 days after pulverization (DAP).

## Data analysis

Data were analyzed by a multivariate statistical method, using factorial analysis. The processes that explained the interactions between the measured variables (surfaces and control) were identified through factorial analysis. After standardization of the variables (null mean and unit variance), the analysis was processed in Statistica 7.0 (StatSoft, Inc., Tulsa, OK, USA).

To extract the factors, the principal component analysis was calculated using the correlation matrix between the variables^34^. Only variables with factor loadings > 0.50 in absolute value were considered. The first factor extracted from the matrix is the linear combination of the original variables, which represents the maximum possible variation contained in the samples. The second factor is defined as the second-best linear combination of variables, subject to the constraint of being orthogonal (independent) to the first factor^35^.

The eigenvalues were higher than unity was considered, according to the criterion established Kairser^36^.The coefficients of the linear functions that define the factor loads were used in the interpretation of their meaning, using the signal and the relative size of the loads as an indication of the weight to be assigned for each variable. Then, the data were subjected to analysis of variance by the F test and presented the treatment averages and standard deviation, for this, the agricolae package was used^37,38^.

## Data Availability

The datasets generated during and/or analyzed during the current study are available from the corresponding author on reasonable request.
